# Restricted infection of xenotropic murine leukemia virus-related virus in human lymphoid tissue

**DOI:** 10.1186/1742-4690-8-S1-A208

**Published:** 2011-06-06

**Authors:** Marta Curriu, Jorge Carrillo, Marta Massanella, Elisabet Garcia, Bonaventura Clotet, Julian Blanco, Cecilia Cabrera

**Affiliations:** 1 Fundación irsiCaixa, Badalona, Barcelona, Spain; 2 Institut d’Investigació en Ciències de la Salut Germans Trias i Pujol, Badalona, Barcelona, Spain

## Background

Xenotropic murine leukemia virus-related virus (XMRV) infects different human cells in vitro. In this study the pathogenic effect of XMRV in lymphoid tissue was investigated by infection of human tonsil histocultures.

## Materials and methods

Human tonsils were obtained from healthy donors, dissected into small blocks and cultured on top of gelfoams. Tissue blocks were left uninfected or infected with an XMRV stock obtained from a 22Rv1 cell culture supernatant in the presence or the absence of AZT or Raltegravir. Viral infection was evaluated by PCR at different times in the cells migrating out the tissue and at day 14 in tissue cells. In addition, tissue cells were immunophenotyped and the expression of XMRV envelope protein was analyzed by western blot.

## Results

Cells migrating out the tissue and tissue cells were positive for XMRV and both AZT and Raltegravir blocked the detection of viral DNA. Despite the presence of XMRV DNA, tissue lysates exhibited undetectable expression of XMRV env proteins by WB. XMRV infection did not modify the percentage of CD3 (63 and 64 in XMRV- and XMRV+ tissue, respectively), CD4 (47 vs 48), CD8 (20 vs 19), or CD19 cells (29 vs 27) neither the naive/memory cell ratio, nor immune activation markers, as evaluated by the expression of HLA-DR and CD38.

## Conclusions

XMRV could specifically infect human lymphoid tissue cells although this process does not culminate in an explicit productive infection. This infection did not result in changes of T or B cells nor in immune activation, suggesting that lymphoid tissue could latently support XMRV infection.

